# HDAC2‐dependent miRNA signature in acute myeloid leukemia

**DOI:** 10.1002/1873-3468.13521

**Published:** 2019-07-19

**Authors:** Mariarosaria Conte, Carmela Dell'Aversana, Giulia Sgueglia, Annamaria Carissimo, Lucia Altucci

**Affiliations:** ^1^ IRCCS, SDN Naples Italy; ^2^ Department of Precision Medicine University of Campania “L. Vanvitelli” Naples Italy; ^3^ Present address: Institute for Applied Mathematics “Mauro Picone” National Research Council Naples Italy

**Keywords:** epigenetics, HDAC2, immunoregulation, leukemia, miRNA, SAHA

## Abstract

Acute myeloid leukemia (AML) arises from a complex sequence of biological and finely orchestrated events that are still poorly understood. Increasingly, epigenetic studies are providing exciting findings that may be exploited in promising and personalized cutting‐edge therapies. A more appropriate and broader screening of possible players in cancer could identify a master molecular mechanism in AML. Here, we build on our previously published study by evaluating a histone deacetylase (*HDAC*)*2*‐mediated miRNA regulatory network in U937 leukemic cells. Following a comparative miRNA profiling analysis in genetically and enzymatically HDAC2‐downregulated AML cells, we identified miR‐96‐5p and miR‐92a‐3p as potential regulators in AML etiopathology by targeting defined genes. Our findings support the potentially beneficial role of alternative physiopathological interventions.

## Graphical abstract

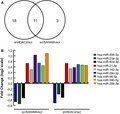

## Abbreviations

**AML**, acute myeloid leukemia

**FC**, fold change

**FDR**, false discovery rate

**HDACi**, histone deacetylase inhibitor(s)

**HDACs**, histone deacetylases

**MHC**, major histocompatibility complex

**SAHA**, suberanilohydroxamic acid

Acute myeloid leukemia (AML) is a multifactorial and highly heterogeneous malignancy, whose incidence rises with age 1. The evolution of the disease is characterized by uncontrolled progenitor cell proliferation and block of differentiation. To date, although many genetic mutations in AML have been identified, prognosis has markedly improved in recent years but still remains poor 2. It is well known that epigenetic mechanisms regulate gene expression and consequently define pathways involved in the physiopathogenesis of AML. Among the many epigenetic regulators, histone deacetylases (HDACs) are tightly involved in AML etiology 3. Notably, *HDAC2* is highly overexpressed in solid and hematological cancers, including AML 4, 5, 6, 7, 8, 9. HDAC2 silencing by enzymatic inhibition has a substantial impact on leukemia cell proliferation and immune regulation, as described in our previous work 10, where we established an *HDAC2*‐knockdown AML clone to better understand the role of cancer cell proliferation dynamics with and without treatment with the well‐studied HDAC inhibitors (HDACi) suberanilohydroxamic acid (SAHA) and entinostat (also known as MS‐275). Several epigenetic drugs, including HDACi, are in fact actively undergoing clinical investigation as single agents or mainly in combination with consolidated chemotherapeutics 11. Similarly, miRNAs are now recognized as epigenetic regulators of transcripts in nearly all physiological processes and human cancers, including AML 12. The key involvement of miRNAs in crucial biological pathways hints at their functional role in complex molecular gene networks in cancer. Recently, the potential use of cellular and circulating miRNAs as biomarkers for AML diagnosis/prognosis, and as therapeutic targets has been widely explored, and many miRNAs were found to be associated with HDAC2 dysfunction in leukemia 13.

Here, we identified a cluster of common up‐ and downregulated miRNAs in both SAHA‐treated and *HDAC2*‐downregulated cells. By miRNA target network computational analysis, we defined an HDAC2‐mediated miRNA signatures in AML by genetic and enzymatic HDAC2 deficiency in a U937 leukemic cell line. We propose a crucial role of miR‐96‐5p and miR‐92a‐3p and related target genes and their relationship with HDAC2 in AML. Here, we corroborated our previous findings and strongly suggested an *HDAC2*‐mediated regulation of the immune system in AML, involving major histocompatibility complex (MHC) class II genes and specific miRNAs, *via* finely tuned molecular mechanisms.

## Materials and methods

### Cell culture and treatment

U937 leukemic cells were kept in RPMI‐1640 medium (EuroClone, Pero, Milan, Italy) supplemented with 10% heat‐inactivated FBS (Gibco, Monza and Brianza, Italy), 100 units·mL^−1^ penicillin G (EuroClone), 100 μg·mL^−1^ streptomycin (EuroClone), 2 mm l‐glutamine (EuroClone), 250 mg·mL^−1^ amphotericin B (EuroClone) and 50 mg·mL^−1^ G‐418 sulfate (Sigma‐Aldrich, Milan, Italy). The cells were incubated at 37 °C at a fixed concentration of CO_2_ (5%). The HDACi SAHA (Merck, Rome, Italy) was dissolved in dimethyl sulfoxide (Sigma‐Aldrich) and used at a final concentration of 5 μm for 6 h of treatment.

### Stable transfection of sh2 vector

Silencing of *HDAC2* in U937 cells was performed as previously described 10.

### RNA isolation and miRNA expression analysis

Total miRNA‐enriched RNA was isolated and miRNA expression levels were analyzed by real‐time PCR as previously reported 14.

### Quantitative real‐time PCR

Real‐time PCR was performed using the VILO cDNA Synthesis Kit (Invitrogen, Monza and Brianza, Italy) to convert RNA into cDNA. 1X SYBR Green PCR Master Mix (BioRad, Segrate, Milan, Italy) was used according to the manufacturer's instructions, using 50 ng of cDNA. Primers used for qRT‐PCR were: *TRIB3* Fw: 5′‐CCAGCTCCTCTACGCCTTTT‐3′ and *TRIB3* Rev: 5′‐CGACACAGCTTGAGATCACG‐3′; *SLC37A3* Fw: 5′‐TGTCCCAGTTCAGCCATCAT‐3′ and *SLC37A3* Rev: 5′‐GAGTCGCTTTCTCTGCACTG‐3′; *TBC1D8* Fw: 5′‐TACTCCTGCTGCTGTTGGAA‐3′ and *TBC1D8* Rev: 5′‐GCTCCTTCTTCTGCGTGGTG‐3′; *FAM49A* Fw: 5′‐GATGGCCAATCG AATGTCCC‐3′ and *FAM49A* Rev: 5′‐ACCATCACCCTCATGCAGAA‐3′. Data were normalized with *GAPDH* Fw: 5′‐GGAGTCAACGGATTTGGTCGT‐3′ and *GAPDH* Rev: 5′‐GCTTCCCGTTCTCAGCCTTGA‐3′.

### miRNA microarray profiling and data analysis

miRNAomes of U937 scramble vector (scr) and HDAC2 knockdown (shHDAC2) cells were analyzed. Each sample was prepared according to Agilent's miRNA Microarray System protocol. Total RNA (100 ng) was dephosphorylated with calf intestine alkaline phosphatase (GE Healthcare Europe, Rome, Italy), denatured with DMSO (Sigma‐Aldrich), and labeled with Cyanin 3‐pCp by T4 RNA ligase (GE Healthcare Europe). The labeled RNA was purified and then hybridized to Human miRNA Microarray (v1) 8x15K (G4470B; Agilent, Cernusco sul Naviglio, Milan, Italy) for 20 h at 55 °C with rotation. After hybridization and washing, the arrays were acquired with an Agilent Scanner and data extracted using agilent feature extraction software (Cernusco sul Naviglio, Milan, Italy), as specified by the manufacturer. Microarray quality control reports generated by the agilent feature extraction software. Using R/BioConductor 15 and limma package, probe level raw intensity was processed. The ‘normexp’ limma method was used for background correction and data normalization was carried out. Differential expression was performed by Student's *t*‐test. The selected miRNA list was obtained by applying a false discovery rate (FDR) < 0.05; each value was converted to log2.

Microarray data are available in the Gene Expression Omnibus (GEO) database (https://www.ncbi.nlm.nih.gov/geo/) under the accession number https://www.ncbi.nlm.nih.gov/geo/query/acc.cgi?acc=GSE129154.

### Computational prediction of miRNA target genes

Target gene prediction of differentially expressed miRNAs was performed using the miRNet database. All miRNA entries are annotated according to the latest miRBase (release 22) (http://mirbase.org/) 16. Target genes were then selected. miRNA target interaction data were downloaded from 11 well‐annotated databases, miRTarBase, TarBase, miRecords, SM2miR, Pharmaco‐miR, miR2Disease, PhenomiR, StarBase, EpimiR, miRDB, and miRanda, selecting the *Homo sapiens* species.

### Gene set enrichment and functional annotation analysis

The relative abundance of ‘Biological Process’ (BP), Pathways (by KEGG), oncogenic and immunologic signatures Gene Ontology terms in each of the selected lists was analyzed using the Molecular Signatures Database v6.2 (MSigDB) in Gene Set Enrichment Analysis (gsea) software (http://software.broadinstitute.org/gsea/msigdb) for Annotation, Visualization and Integrated Discovery.

### Gene expression microarray profiling and data analysis using the Agilent platform

Gene expression profiles of U937 scramble vector (scr) and HDAC2 knockdown (shHDAC2) cells were analyzed by Whole Human Genome Two‐Color Microarray (G4112F; Agilent), following the manufacturer's protocol. Microarray data are available in the Gene Expression Omnibus (GEO) database (http://www.ncbi.nlm.nih.gov/gds) under the accession number https://www.ncbi.nlm.nih.gov/geo/query/acc.cgi?acc=GSE37529 10. Probe‐level raw intensity was processed using R/BioConductor and limma package. Background correction was performed using ‘normexp’ limma method and data normalization was carried out in two steps: LOWESS normalization within array to correct systematic dye bias and quantile normalization between arrays to detect systematic nonbiological bias. Ratios representing the relative target mRNA intensities compared to control RNA probe signals were derived from normalized data. For each *P*‐value, the Benjamini–Hochberg procedure was used to calculate the FDR in order to avoid the problem of multiple testing.

## Results

### Differentially expressed miRNA profiling in HDAC2‐defective AML

**Figure 1:**
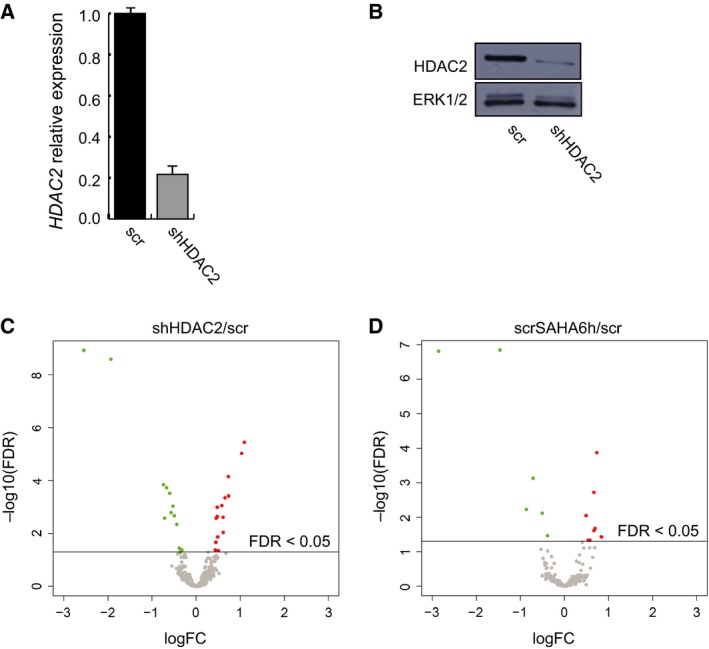
Differentially expressed miRNAs in a validated HDAC2‐defective AML clone. (A) qRT‐PCR validation in shHDAC2 clone compared to scr control. Data represent mean values from three parallel experiments with error bars showing standard deviations above each column. (B) Western blot analysis of HDAC2 in shHDAC2 and scr clones. Normalization was performed with ERK1/2. (C) Volcano plot showing differentially expressed miRNAs in shHDAC2 compared to scr cells. Student's *t*‐test analysis identified 29 differentially expressed miRNAs by applying an FDR significance threshold < 0.05. (D) Volcano plot showing differentially expressed miRNAs in scrSAHA6h compared to scr cells. Student's *t*‐test analysis identified 14 differentially expressed miRNAs by applying an FDR significance threshold < 0.05.

**Figure 2:**
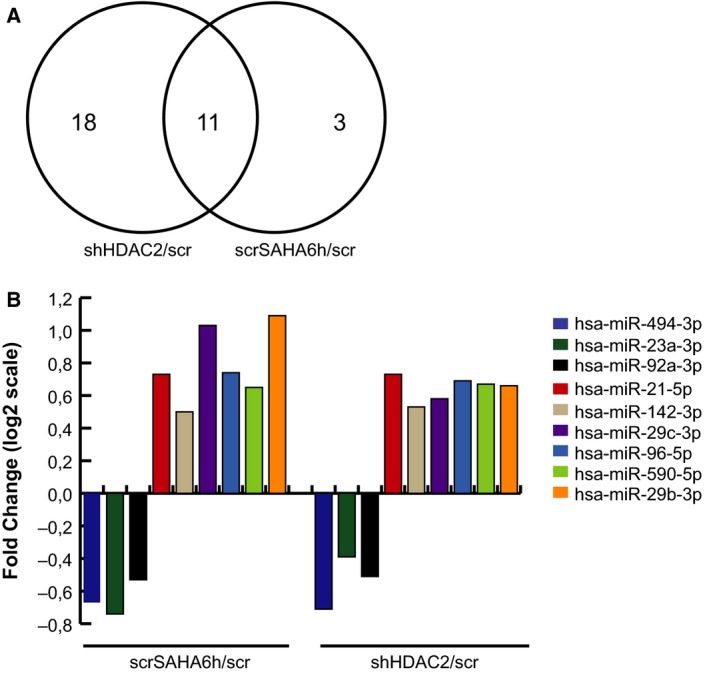
Comparative analysis of commonly regulated miRNAs. (A) Venn diagram showing the intersection of differentially expressed miRNAs in shHDAC2/scr and scrSAHA6h/scr cells. Comparative analysis showed that 11 miRNAs are commonly regulated both when HDAC2 is genetically silenced and enzymatically inhibited. (B) Microarray expression FC in log2 of nine miRNAs altered in scrSAHA6h/scr and shHDAC2/scr cells. Data show three downregulated and six upregulated miRNAs in both conditions.

We built on our previous epigenetic study 10 by evaluating the impact of HDAC2 deficiency on miRNA expression in AML. miRNome analysis was performed in HDAC2‐silenced (shHDAC2) and relative scramble control (scr) U937 stable clones, previously obtained and retested for mRNA and HDAC2 protein expression levels (Fig. 1A,B). In addition, we treated scr cells with the well‐known HDACi SAHA for 6 h (scrSAHA6h) to enzymatically mimic HDAC2 silencing. Volcano plots display differentially expressed miRNAs in shHDAC2 and scrSAHA6h compared to scr cells (Fig. 1C,D). Student's *t*‐test analysis revealed the presence of 29 and 14 differentially expressed miRNAs in shHDAC2/scr and scrSAHA6h/scr cells, respectively, by applying an FDR significance threshold < 0.05 (Tables 1 and 2). Comparative analysis showed that 11 miRNAs are commonly regulated both when HDAC2 is genetically silenced and enzymatically inhibited (Fig. 2A). Among these, miR‐801 and miR‐923 were excluded because they were removed from the miRBase (v22) 16; miR‐801 appears to be a fragment of U11 spliceosomal RNA, while miR‐923 seems to be a fragment of the 28S rRNA. The nine miRNAs altered (three downregulated and six upregulated) in each HDAC2‐defective condition are shown in Fig. 2B. We speculate that this cluster of miRNAs may suggest an HDAC2‐dependent miRNA signature.

**Table 1:** Twenty‐nine differentially expressed miRNAs in shHDAC2 vs scr AML cells identified by applying an FDR significance threshold < 0.05.

miRNA shHDAC2 vs scr	FDR	Fold_Change
hsa‐miR‐801_v10.1	1.19E‐09	−2.543737874
hsa‐miR‐923_v12.0	2.60E‐09	−1.933159241
hsa‐miR‐23a	0.000143328	−0.739889068
hsa‐miR‐455‐3p	0.002630851	−0.713125392
hsa‐miR‐494	0.000185858	−0.666803687
hsa‐miR‐221	0.000303258	−0.598941562
hsa‐miR‐378	0.001634909	−0.568655832
hsa‐miR‐92a	0.000922419	−0.52700539
hsa‐miR‐362‐5p	0.002131758	−0.495345929
hsa‐miR‐30d	0.004568985	−0.438824481
hsa‐miR‐155	0.035382109	−0.381530837
hsa‐miR‐140‐3p	0.049756326	−0.354392812
hsa‐miR‐130b	0.041388762	−0.321803064
hsa‐miR‐25	0.042613013	−0.320835912
hsa‐miR‐101	0.041951895	0.435021196
hsa‐miR‐21*	0.021548756	0.448826916
hsa‐miR‐27b	0.002648152	0.456534869
hsa‐let‐7i	0.001031153	0.479164548
hsa‐miR‐22	0.002282868	0.480444244
hsa‐miR‐324‐5p	0.013534385	0.491241024
hsa‐miR‐142‐3p	0.044475072	0.50184352
hsa‐miR‐30e	0.000884468	0.58335757
hsa‐miR‐142‐5p	0.002423095	0.611920185
hsa‐miR‐340	0.009075971	0.61480908
hsa‐miR‐590‐5p	0.000446421	0.654156226
hsa‐miR‐21	7.14E‐05	0.733582232
hsa‐miR‐96	0.00038477	0.737484019
hsa‐miR‐29c	9.38E‐06	1.032982453
hsa‐miR‐29b	3.58E‐06	1.094410243

**Table 2:** Fourteen differentially expressed miRNAs in scrSAHA6h vs scr AML cells identified by applying an FDR significance threshold < 0.05.

miRNA scrSAHA6h vs scr	FDR	Fold_Change
hsa‐miR‐801_v10.1	1.53E‐07	−2.8559
hsa‐miR‐923_v12.0	1.42E‐07	−1.46423
hsa‐miR‐19b‐1*	0.005977	−0.86651
hsa‐miR‐494	0.00074	−0.71079
hsa‐miR‐92a	0.007581	−0.50668
hsa‐miR‐23a	0.034233	−0.38589
hsa‐miR‐148a	0.008932	0.489832
hsa‐miR‐142‐3p	0.046551	0.532725
hsa‐miR‐29c	0.04658	0.576009
hsa‐miR‐29b	0.001918	0.664701
hsa‐miR‐590‐5p	0.024333	0.665683
hsa‐miR‐96	0.021213	0.694239
hsa‐miR‐21	0.000133	0.733582
hsa‐miR‐210	0.037251	0.835821

### miRNA target networks and enrichment analysis

**Figure 3:**
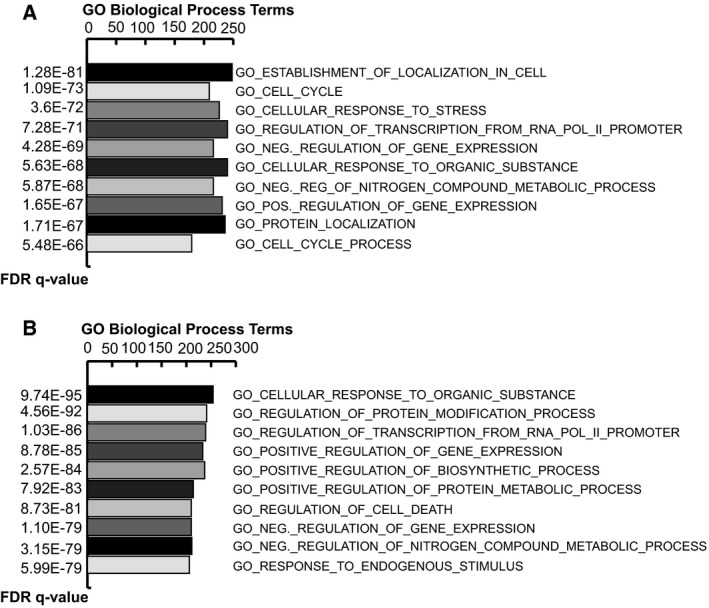
Gene Ontology Enrichment of HDAC2‐dependent miRNA target genes. (A) Functional annotation chart (BP‐GO biological process) of predicted target genes of three downregulated miRNAs in shHDAC2/scr and scrSAHA6h/scr cells obtained from MSigDB using gsea software. (B) Functional annotation chart (BP‐GO biological process) of predicted target genes of six upregulated miRNAs in shHDAC2/scr and scrSAHA6h/scr cells obtained from MSigDB using gsea software.

To predict miRNA targets, we interrogated miRNet (https://www.mirnet.ca/) 17 and identified 1711 predicted targets of the three downregulated miRNAs (Table S1), and 1418 predicted targets of the six upregulated miRNAs (Table S2). To investigate the biological functions, regulatory mechanisms, and disease relevance of differentially expressed HDAC2‐dependent miRNAs and their relative target genes, we used MSigDB (v6.2) software applying an FDR *q*‐value significance threshold < 0.001. Figure 3A,B shows the biological processes of predicted target genes of the three downregulated and six upregulated miRNAs in shHDAC2/scr and scrSAHA6h/scr cells, respectively. HDAC2 dysfunction in AML cells after both genetic and enzymatic downregulation is in line with the biological processes in terms of cell cycle regulation, cell and protein localization, and response to organic substance (i.e., the HDACi SAHA). We also looked for the immunologic signature, consisting of gene sets representing cell types, conditions and alterations within the immune system, in predicted target genes of the three downregulated miRNAs (Table 3) and six upregulated miRNAs (Table 4). As shown in Tables 3 and 4, many genes involved in immunoregulatory mechanisms are perturbed, further confirming our previous finding that the immune system is affected in an HDAC2‐defective AML clone (in both genetic and enzymatic conditions).

**Table 3:** Immunologic signatures of predicted target genes of three downregulated miRNAs identified by applying an FDR *q*‐value significance threshold ≤ 0.001.

Gene set name	No. of genes in gene set (*K*)	No. of genes in overlap (*k*)	*k*/*K*	*P*‐value	FDR *q*‐value
GSE2405_0H_VS_9H_A_PHAGOCYTOPHILUM_STIM_NEUTROPHIL_DN	200	59	0.295	1.42E‐37	6.93E‐34
GSE9006_HEALTHY_VS_TYPE_1_DIABETES_PBMC_1MONTH_POST_DX_UP	200	56	0.28	2.18E‐34	5.31E‐31
GSE2405_0H_VS_24H_A_PHAGOCYTOPHILUM_STIM_NEUTROPHIL_UP	200	51	0.255	2.68E‐29	4.35E‐26
GSE39820_TGFBETA1_VS_TGFBETA3_IN_IL6_TREATED_CD4_TCELL_UP	200	48	0.24	2.21E‐26	2.15E‐23
GSE9006_HEALTHY_VS_TYPE_1_DIABETES_PBMC_4MONTH_POST_DX_UP	200	48	0.24	2.21E‐26	2.15E‐23
GSE27241_WT_VS_RORGT_KO_TH17_POLARIZED_CD4_TCELL_TREATED_WITH_DIGOXIN_UP	170	44	0.2588	9.27E‐26	7.53E‐23
GSE17721_CTRL_VS_POLYIC_4H_BMDC_UP	200	46	0.23	1.69E‐24	1.17E‐21
GSE1460_DP_THYMOCYTE_VS_NAIVE_CD4_TCELL_CORD_BLOOD_UP	200	45	0.225	1.41E‐23	6.27E‐21
GSE2770_IL4_ACT_VS_ACT_CD4_TCELL_2H_UP	200	45	0.225	1.41E‐23	6.27E‐21
GSE41978_ID2_KO_VS_ID2_KO_AND_BIM_KO_KLRG1_LOW_EFFECTOR_CD8_TCELL_DN	200	45	0.225	1.41E‐23	6.27E‐21

**Table 4:** Immunologic signatures of predicted target genes of six upregulated miRNAs in AML cells identified by applying an FDR *q*‐value significance threshold ≤ 0.001.

Gene set name	No. of genes in gene set (*K*)	No. of genes in overlap (*k*)	*k*/*K*	*P*‐value	FDR *q*‐value
GSE42021_TREG_PLN_VS_CD24INT_TREG_THYMUS_UP	200	50	0.25	5.54E‐32	2.70E‐28
GSE27434_WT_VS_DNMT1_KO_TREG_DN	200	47	0.235	7.55E‐29	1.84E‐25
GSE22025_PROGESTERONE_VS_TGFB1_AND_PROGESTERONE_TREATED_CD4_TCELL_UP	199	43	0.2161	6.19E‐25	1.01E‐21
GSE14769_UNSTIM_VS_60MIN_LPS_BMDM_DN	200	42	0.21	7.15E‐24	5.81E‐21
GSE20500_RETINOIC_ACID_VS_RARA_ANTAGONIST_TREATED_CD4_TCELL_DN	200	42	0.21	7.15E‐24	5.81E‐21
GSE39820_TGFBETA3_IL6_VS_TGFBETA3_IL6_IL23A_TREATED_CD4_TCELL_UP	200	42	0.21	7.15E‐24	5.81E‐21
GSE3920_IFNA_VS_IFNG_TREATED_ENDOTHELIAL_CELL_UP	166	38	0.2289	3.76E‐23	2.61E‐20
GSE13411_SWITCHED_MEMORY_BCELL_VS_PLASMA_CELL_UP	200	41	0.205	6.47E‐23	3.50E‐20
GSE4748_CTRL_VS_LPS_STIM_DC_3H_UP	200	41	0.205	6.47E‐23	3.50E‐20
GSE12003_MIR223_KO_VS_WT_BM_PROGENITOR_4D_CULTURE_UP	200	40	0.2	5.68E‐22	1.97E‐19

### Identification of HDAC2‐dependent miRNA targets

**Figure 4:**
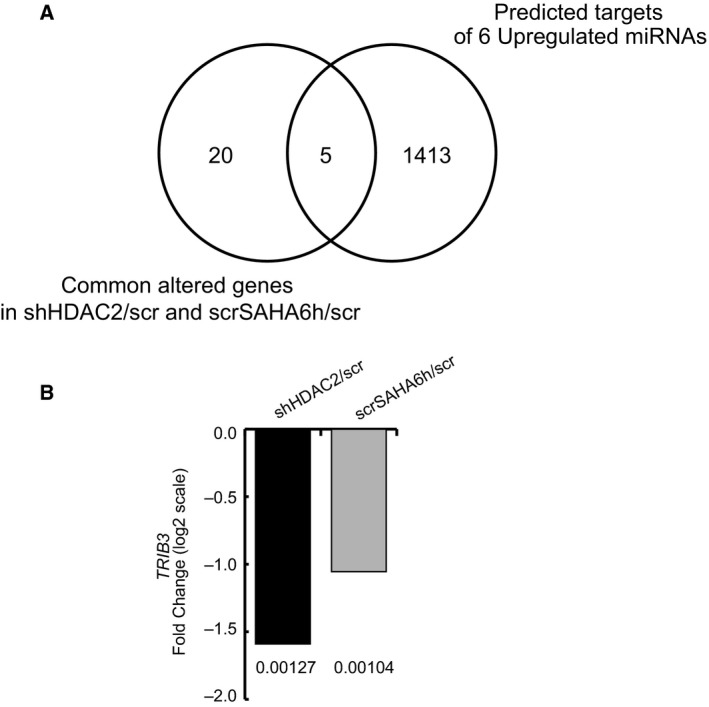
Intersection analysis between commonly altered genes in shHDAC2/scr and scrSAHA6h/scr cells and predicted targets of upregulated miRNAs. (A) Venn diagram showing the five common altered target genes of six upregulated miRNAs in shHDAC2/scr and scrSAHA6h/scr cells, also predicted by computational analysis. (B) Microarray expression FC in log2 in shHDAC2 and scrSAHA6h cells compared to scr cells for *TRIB3* downregulated hit targeted by miR‐96‐5p. The FDR are reported on histogram bars.

**Figure 5:**
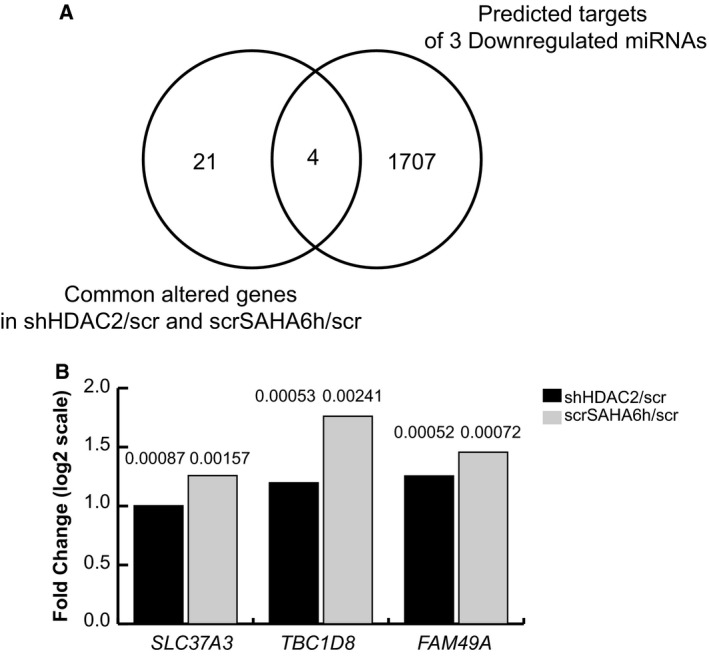
Intersection analysis between commonly altered target genes in shHDAC2/scr and scrSAHA6h/scr cells associated with downregulated miRNAs. (A) Venn diagram showing the four common altered target genes of three downregulated miRNAs in shHDAC2/scr and scrSAHA6h/scr cells, also predicted by computational analysis. (B) Microarray expression FC in log2 in shHDAC2 and scrSAHA6h cells compared to scr cells for *SLC37A3*,* TBC1D8*, and *FAM49A* upregulated hits targeted by miR‐92a‐3p. The FDR are reported on histogram bars.

To validate target prediction analysis, we performed a very stringent intersection analysis between the commonly altered genes in shHDAC2/scr and scrSAHA6h/scr cells (https://www.ncbi.nlm.nih.gov/geo/query/acc.cgi?acc=GSE37529) 10 (Table 5) and the predicted miRNA hits. Among the targets of the six upregulated miRNAs, we identified five predicted gene targets (*TRIB3*,* SLC37A3*,* EMP1*,* SCD*,* IL1B*) also regulated in gene expression profiles of shHDAC2/scr and scrSAHA6h/scr cells (Fig. 4A). Only one downregulated hit corresponding to *TRIB3* gene displayed an according trend compared to the related regulating miRNA, mir‐96‐5p. Figure 4B shows *TRIB3* microarray expression fold change (FC) in log2 in shHDAC2 and scrSAHA6h compared to scr cells. In contrast, distinguishing between the targets of the three downregulated miRNAs, we identified four predicted gene targets (*SLC37A3*,* TBC1D8*,* SCD*,* FAM49A*) regulated in gene expression profiles of shHDAC2/scr and scrSAHA6h/scr cells (Fig. 5A). *SLC37A3*,* TBC1D8*, and *FAM49A* are upregulated hits targeted by miR‐92a‐3p. Figure 5B shows the microarray expression FC in log2 of three upregulated target genes in both shHDAC2 and scrSAHA6h compared to scr cells. *SCD* was excluded as it showed a different trend in the two conditions (FC = −1.03 in scrSAHA6h/scr; FC = 1.46 in shHDAC2/scr). These data are in line with trends in mRNA regulation, target prediction, and miRNA expression levels.

**Table 5:** Genes commonly altered in shHDAC2/scr and scrSAHA6h/scr AML cells identified by applying an FDR significance threshold < 0.05.

Gene symbol
*ANXA1*
*ARHGEF3*
*BMF*
*CX3CR1*
*EMP1*
*EPAS1*
*FAM117A*
*FAM49A*
*FPR1*
*GRB10*
*H1F0*
*HLA‐DMB*
*IL1B*
*IL4I1*
*LOH11CR2A*
*LPAAT‐THETA*
*MAFB*
*MMP1*
*RNF149*
*SCD*
*SLC37A3*
*SYTL3*
*TBC1D8*
*TMEM118*
*TRIB3*

### Validation of miRNAs and target genes in HDAC2‐defective U937 cells

**Figure 6:**
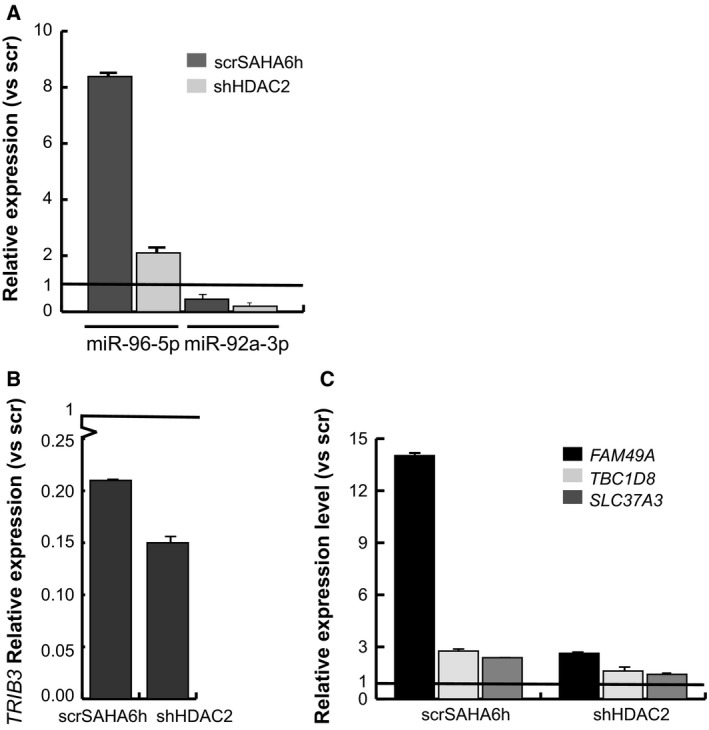
Analysis of expression levels of miRNAs and corresponding target genes in U937 HDAC2‐defective clone. (A) qRT‐PCR of miR‐92a‐3p and miR‐96‐5p. Expression levels were evaluated in U937 shHDAC2, scr, and scrSAHA6h cells. Data represent mean values from three parallel experiments with error bars showing standard deviations above each column. (B) qRT‐PCR of *TRIB3* miR‐96‐5p‐target gene. Expression levels were evaluated in U937 shHDAC2, scr, and scrSAHA6h cells. Data represent mean values from three parallel experiments with error bars showing standard deviations above each column. (C) qRT‐PCR of *SLC37A3*,* FAM49A*, and *TBC1D8* miR‐92a‐3p‐target genes. Expression levels were evaluated in U937 shHDAC2, scr, and scrSAHA6h cells. Data represent mean values from three parallel experiments with error bars showing standard deviations above each column.

Following miRNA microarray profiling and computational prediction of miRNA target genes, we investigated the expression levels of miRNAs and their corresponding target genes. We analyzed miR‐92a‐3p and miR‐96‐5p expression levels by real‐time PCR (Fig. 6A). Gene expression levels of *TRIB3*, a target of miR‐96‐5p, were analyzed in scr and shHDAC2 cells as well as in scr cells untreated or treated with SAHA for 6 h. *TRIB3* relative expression was downregulated in both HDAC2‐deficient and scr U937 cells treated with SAHA, suggesting a correlated response due to HDAC2 enzymatic inhibition and genetic silencing (Fig. 6B). The expression levels of *SLC37A3, FAM49A,* and *TBC1D8*, target genes of hsa‐miR‐92a‐3p, were also analyzed (Fig. 6C). According to miRNA expression, all target genes were upregulated in shHDAC2 as well as in scrSAHA treated cells, related to scr. Notably, miRNA‐mRNA regulation in HDAC2‐downregulated cells was comparable after both enzymatic and pharmacological inhibition by SAHA, supporting the hypothesis that upregulated expression levels of HDAC2 indicate dysfunction of these regulators in AML.

## Discussion

miRNAs are known to play a critical and functional role in a broad range of key molecular processes *via* sophisticated regulation of distinct targets, orchestrating a molecular intracellular balance of gene expression. miRNA activity and expression are affected in cancer. Altered miRNAs in AML are involved in a variety of biological pathways 18, and a better understanding of their signatures might help unravel the complexity associated with the emergence of this disease. Since HDAC2 deregulation affects cell proliferation, apoptosis, and immune system in AML, focusing on specific miRNAs altered by this epigenetic regulator may identify potential markers for determining the best strategies in AML treatment. To date, many miRNAs were found directly targeted by HDAC2 in several cancers such as colorectal cancer 19, hepatocellular carcinoma 20, breast cancer 21, and AML 22. In AML, differentially expressed miRNAs have a prognostic and functional role associated with cytogenetics, molecular features, molecular markers, morphology, and clinical outcome 23. In a previous study, we found that *HDAC2* gene is considerably upregulated in AML *ex vivo* patient samples and cell lines. Following HDAC2 silencing and enzymatic inhibition using the epigenetic‐based drug SAHA, we observed a pivotal *HDAC2*‐dependent modulation of chromatin architecture leading to transcriptional changes promoting mainly activation of an immune response. Specifically, HDAC2 acts directly at epigenetic level by regulating the promoter regions of specific allelic forms of MHC class II genes (*HLA‐DRA* and *HLA‐DPA1*). Here, based also on our previous findings, we elucidated miRNA‐HDAC2 crosstalk and its involvement in AML state. Interestingly, a stringent computational analysis between the transcriptome and miRNome profiles in shHDAC2/scr and scrSAHA6h/scr cells identified a crucial role for miR‐96‐5p and miR‐92a‐3p, and defined their target gene regulation enclosing convergent pathways already identified in our previous work. We investigated upregulated miR‐96‐5p, which has an oncogenic role in several cancer types 24, 25, 26. Low expression levels of miR‐96‐5p were found in a specific cohort of AML patients, suggesting that this epigenetic marker could be considered a prognostic factor for AML at diagnosis 27. miR‐96‐5p upregulation acts as an antiapoptotic factor in bladder cells, as it negatively regulates specific targets such as *CDKN1A*, which is involved in cell cycle regulation and DNA damage pathway in bladder cancer 28. Our data show that *TRIB3* is targeted by miR‐96‐5p. The protein encoded by *TRIB3* gene is a potential kinase that negatively regulates NF‐kB and Akt1 pathways, affecting cell proliferation and apoptosis, and promoting ubiquitination‐dependent degradation of several key proteins. *TRIB3* is strongly expressed in AML with t(8;21) and t(15;17) translocations, as well as in M2/M3 AML subtypes 29, although its specific role in leukemogenesis is still elusive. However, evidence suggests that *TRIB3* contributes to acute promyelocytic leukemia progression by PML‐RARα stabilization *via* specific binding to SUMOylation motifs, thereby acting on PML‐RARα degradation and differentiation 30. In addition, we found and subsequently investigated the downregulation of miR‐92a‐3p targeting *SLC37A3*,* TBC1D*, and *FAM49A* genes. High expression levels of miR‐92a‐3p are associated with acute megakaryoblastic leukemia and affect genes controlling apoptosis and cell proliferation 31. High expression levels of this miRNA were also found in both AML and acute lymphoblastic leukemia cells compared to normal blasts 32. We identified *SLC37A3* as one of the targets of miR‐92a‐3p. This transmembrane protein is localized in the endoplasmatic reticulum and is involved in sugar transport. The *SLC37A3* gene is one of the four sugar‐phosphate exchanger family members, but its functional activity is not yet clear. Evidence suggests that *SLC37A3* might be involved in physiopathological regulation in pancreatic but also in immune system 33. The methylation levels of this gene affect glucose blood degree, suggesting its potential role in epigenetic modifications *via* a mechanism that still requires further investigation, and its involvement in obesity‐related metabolism. We also identified *FAM49A* as a miR‐92a‐3p‐target by computational analysis. This target gene was detected as a downregulated protein in bladder cancer cells 34. *FAM49A* is a consensus PU.1‐activated target gene. PU.1 is an E26 transformation‐specific family transcription factor widely involved in hematopoiesis. *FAM49A* is a direct functional regulator of myeloid, dendritic cell, B cell and a differentiation factor of earliest stages of T‐cell and terminal erythroid cell 35. The third hsa‐miR‐92a‐3p target which we investigated is *TBC1D8*. This gene is a member of the Tre2/Bub2/Cdc16 (TBC) domain protein family, characterized by highly conserved TBC domains 36. *TBC1D8* was found among differentially expressed genes in pre‐B acute lymphocytic leukemia samples with ALL1/AF4, E2A/PBX1, and BCR/ABL molecular rearrangements, and positively controls cell proliferation 37. Other studies identified *TBC1D8* as a target of *IL4* in chronic lymphocytic leukemia and normal B cells 38. In this work, we propose the existence of a mechanistic crosstalk between miRNAs and *HDAC2* in an epigenetic superstructure regulating pathogenesis and progression of AML. All our findings converge in identifying *HDAC2* and miRNA interplay in specific biological processes (Fig. 6), which potentially affects regulation of gene expression, cell cycle, apoptosis, response to stress and response to organic substance. These mechanisms are robustly altered in leukemogenesis, further confirming our previous findings. We mostly speculate on the immunologic signature of predicted target genes of both downregulated and upregulated miRNAs (Tables 4 and 5) identifies immune cells such as CD4 and CD8T in a specific gene set. It is not surprising that some hits were also associated with type I diabetes, as MHC class II genes are related to this disease 39. Since MHC class II genes regulate initiation of immune response, our data characterize a specific signature also involving endothelial, thymic, epithelial, and B cells in gene sets. The epigenetic drug SAHA was used as a therapeutic agent in this and in our previous study. This drug plays a critical role as an immunomodulating agent by enhancing cancer cell immunogenicity. We and other authors reported that HDACi make cancer cells more responsive to immunotherapy by increasing the expression levels of tumor antigens, and drive gene expression toward a proapoptotic mechanism in cancer 40, 41. Finally, taken together, our findings identified miR‐96‐5p and miR‐92a‐3p as prospective epi‐regulators in AML. This *HDAC2*‐dependent miRNA signature in AML highlights the potentially beneficial effects of treatment with epigenetic drugs alone or in combination with other therapies (including immunotherapy) acting *via* a targeted mechanism involving the perturbation of genes affecting cell cycle, proliferation, apoptosis, and immune system. To date, achieving greater insights into leukemogenesis has allowed us to make progress toward the prevention and treatment of this devastating disease. Given that many disease agents including those that are not strictly biological (such as smoking, obesity, exposure to certain types of radiation or other substances) are not always controllable and vary continuously throughout a person's lifetime, the need for multifaceted therapeutic approaches is imperative.

## Author contributions

LA supervised the study; MC and CD designed, performed experiments, and wrote the manuscript; GS performed experiments; AC analyzed GE and miRNA data.

## Supporting information


**Table S1.** Predicted targets of three downregulated miRNAs obtained from miRNet database (n = 1711).
**Table S2.** Predicted targets of six upregulated miRNAs obtained from miRNet database (n = 1418).
